# Biochemical and genetic characteristics of patients with primary carnitine deficiency identified through newborn screening

**DOI:** 10.1186/s13023-021-02126-3

**Published:** 2021-12-04

**Authors:** Yiming Lin, Bangbang Lin, Yanru Chen, Zhenzhu Zheng, Qingliu Fu, Weihua Lin, Weifeng Zhang

**Affiliations:** 1 Center of Neonatal Disease Screening, Quanzhou Maternity and Children’s Hospital, 700 Fengze Street, Quanzhou, 362000 Fujian Province China; 2 Administrative Office, Quanzhou Maternity and Children’s Hospital, 700 Fengze Street, Quanzhou, 362000 Fujian Province China; 3 Department of Neonatology, Quanzhou Maternity and Children’s Hospital, 700 Fengze Street, Quanzhou, 362000 Fujian Province China

**Keywords:** Primary carnitine deficiency, Newborn screening, *SLC22A5* gene, Free carnitine, Tandem mass spectrometry

## Abstract

**Background:**

Primary carnitine deficiency (PCD) is an autosomal recessive disorder of carnitine transportation that leads to impaired fatty acid oxidation. Large-scale studies on newborn screening (NBS) for PCD are limited. This study aimed to investigate the biochemical and genetic characteristics of patients with PCD detected through NBS.

**Results:**

A total of 548 247 newborns were screened for PCD between January 2014 and June 2021; 1714 newborns with low free carnitine (C0) levels were called back and 49 patients were diagnosed with PCD. The latest incidence rate in Quanzhou, China, was estimated to be 1 in 11 189 newborns. NBS results showed that the 49 patients had varying degrees of decreased C0 levels, whereas seven patients exhibited normal C0 levels during the recall review. All patients harbored biallelic pathogenic variants of the *SLC22A5* gene. Nineteen distinct *SLC22A5* variants were detected in these 49 patients, and most of the detected variants were clustered in exons 1, 4, and 7. The top eight variants had an allele frequency of 86.73%. The most common variant was c.760C > T (p.R254*) with an allele frequency of 31.63%, followed by c.51C > G (p.F17L) (17.35%) and c.1400C > G (p.S467C) (16.33%). The C0 level of patients with the N/N genotype was significantly lower than that of the M/M group. The C0 levels of patients with genotypes of R254*/R254* and R254*/F17L were far lower than those of patients with the R254*/S467C genotype.

**Conclusions:**

This study presented more than 500,000 NBS data with the latest incidence of 1:11 189 in the Quanzhou area. The *SLC22A5* variant spectrum in the selected southern Chinese population has been updated. Patients with null variants were associated with low C0 levels. Combining NBS with genetic testing is critical to improve screening efficiency because patients with PCD may have normal C0 levels during NBS and recall review.

## Background

Primary carnitine deficiency (PCD, OMIM #212140) is an autosomal recessive disorder of carnitine transportation that leads to impaired fatty acid oxidation. The causative gene, *SLC22A5* (MIM# 603377), was mapped to chromosome 5q31 and encodes the organic cation transporter novel 2 (OCTN2) [1, 2]. Patients with this defect experience continuous renal loss of carnitine, defective ketogenesis, and low serum carnitine levels, resulting in muscle weakness and pain, as well as cardiomyopathy and arrhythmias. The clinical presentation of PCD shows obvious variability ranging from no clinical symptoms to acute metabolic decompensation early in life, progressive hypertrophic cardiomyopathy later in life, or even sudden death from cardiac arrhythmia [3–6]. Lifelong high-dose carnitine supplementation is recommended for patients with PCD regardless of the severity of presentation, and the long-term prognosis is favorable with timely treatment [7].

Newborn screening (NBS) is a useful preventive health measure for early diagnosis and has been demonstrated to be economically efficient. Given that PCD is a well-treatable metabolic disease and adverse symptoms can be prevented, PCD has been included in many NBS panels worldwide. NBS for PCD was performed using tandem mass spectrometry (MS/MS), by measuring free carnitine (C0) levels below the cut-off value in dried blood spot samples. The diagnosis of PCD after NBS is confirmed by genetic analysis of the *SLC22A5* gene or by verifying reduced carnitine transport activity in fibroblasts [8, 9]. The measurement of carnitine transport activity is a reliable diagnostic assay, but it is only performed in a few laboratories worldwide and requires a skin biopsy. Therefore, genetic testing is mainly used for diagnostic confirmation. However, it is noteworthy that some disease-causing pathogenic variants can escape detection because the genetic diagnostic yield for PCD has been relatively low [10].

Although several reports of PCD NBS in the Chinese population have been reported, the screened number and confirmed positive patients are relatively limited [11–13]. The expanded NBS program was implemented in Quanzhou, China, in January 2014. To date, more than 500,000 newborns have been screened for PCD. In this study, we report our experience with NBS for PCD over 8 years in southern China. We sought to determine the latest incidence, biochemical, and genetic features of PCD identified using NBS. Additionally, we investigated the relationship between the genotype and biochemical phenotype of patients with PCD.

## Results

### NBS for PCD

Overall, 548 247 newborns were screened for PCD during the study period. NBS results showed that 1714 newborns had low C0 levels, yielding a positivity rate of 0.31% (1714/548 247). Forty-nine patients with biallelic pathogenic variants in *SLC22A5* were diagnosed with PCD, and the positive predictive value was approximately 2.86% (49/1714). Consequently, the incidence rate in Quanzhou was estimated to be 1 in 11 189 newborns. In addition, six maternal patients with PCD were identified because of low C0 levels in their infants.

### Biochemical features

The initial NBS results showed that 49 patients had varying degrees of decreased C0 levels. The mean C0 level at NBS in this cohort was 4.16 ± 1.78 μmol/L (range 1.63–8.25, cut-off value: 8.5–50 μmol/L). In comparison, seven patients (14.3%) (nos. 15, 16, 26, 27, 30, 37, and 42) exhibited normal C0 levels during the recall review. The mean C0 level was increased to 4.69 ± 3.01 μmol/L on the second screen (range 1.05–14.27, cut-off value: 8.5–50 μmol/L) (Table 1).

**Table 1:** Biochemical and genetic characteristics of 49 patients with primary carnitine deficiency (PCD)

Patient no	Gender	C0	C0-F1	Genotype		References
1	Male	4.61	6.29	c.51C > G (p.F17L)	c.1195C > T(p.R399W)	This study
2	Female	3.28	3.17	c.51C > G (p.F17L)	c.51C > G (p.F17L)	This study
3	Female	2.37	1.05	c.338G > A (p.C113Y)	c.760C > T (p.R254*)	This study
4	Male	7.65	5.04	c.51C > G (p.F17L)	c.1400C > G (p.S467C)	This study
5	Male	3.75	3.19	c.760C > T (p.R254*)	c.1400C > G (p.S467C)	This study
6	Male	2.72	2.86	c.844C > T (p.R282*)	c.1400C > G (p.S467C)	This study
7	Female	2.54	2.29	c.695C > T (p.T232M)	c.760C > T (p.R254*)	This study
8	Male	5.29	6.58	c.51C > G (p.F17L)	c.1195C > T(p.R399W)	This study
9	Female	5.02	5.53	c.428C > T (p.P143L)	c.428C > T (p.P143L)	This study
10	Female	1.63	1.67	c.760C > T (p.R254*)	c.760C > T (p.R254*)	This study
11	Male	2.31	2.76	c.51C > G (p.F17L)	c.1161 T > G(p.Y387*)	This study
12	Female	3.49	3.52	c.51C > G (p.F17L)	c.760C > T (p.R254*)	Lin et al. [10]
13	Male	1.96	1.73	c.51C > G (p.F17L)	c.760C > T (p.R254*)	Lin et al. [21]
14	Female	2.40	1.44	c.760C > T (p.R254*)	c.760C > T (p.R254*)	Lin et al. [21]
15	Male	5.78	**10.67**	c.760C > T (p.R254*)	c.797C > T (p.P266L)	Lin et al. [21]
16	Male	5.95	**8.64**	c.695C > T (p.T232M)	c.1160A > G (p.Y387C)	Lin et al. [21]
17	Female	7.27	6.66	c.760C > T (p.R254*)	c.797C > T (p.P266L)	Lin et al. [21]
18	Female	5.58	5.59	c.760C > T (p.R254*)	c.1400C > G (p.S467C)	Lin et al. [21]
19	Female	5.34	6.02	c.797C > T (p.P266L)	c.394-1G > A	Lin et al. [21]
20	Female	1.78	1.90	c.695C > T (p.T232M)	c.1139C > T (p.A380V)	Lin et al. [21]
21	Male	4.34	4.45	c.51C > G (p.F17L)	c.51C > G (p.F17L)	Lin et al. [21]
22	Female	4.75	4.16	c.760C > T (p.R254*)	c.845G > A (p.R282Q)	Lin et al. [21]
23	Female	3.45	5.24	c.760C > T (p.R254*)	c.1400C > G (p.S467C)	Lin et al. [21]
24	Female	6.82	5.02	c.760C > T (p.R254*)	c.1400C > G (p.S467C)	Lin et al. [21]
25	Male	2.19	2.12	c.822G > A (p.W274*)	c.782_799del ((p.V261_P266del)	Lin et al. [21]
26	Male	2.73	**9.84**	c.51C > G (p.F17L)	c.1144_1162del (p.V382Cfs*45)	Lin et al. [21]
27	Male	3.00	**10.81**	c.51C > G (p.F17L)	c.1400C > G (p.S467C)	Lin et al. [21]
28	Male	6.46	5.10	c.695C > T (p.T232M)	c.1400C > G (p.S467C)	Lin et al. [21]
29	Male	3.02	1.77	c.760C > T (p.R254*)	c.760C > T (p.R254*)	Lin et al. [21]
30	Female	6.77	**10.05**	c.1400C > G (p.S467C)	c.1400C > G (p.S467C)	Lin et al. [21]
31	Female	2.36	1.75	c.760C > T (p.R254*)	c.760C > T (p.R254*)	Lin et al. [21]
32	Female	3.12	2.88	c.760C > T (p.R254*)	c.51C > G (p.F17L)	Lin et al. [21]
33	Male	3.64	3.80	c.695C > T (p.T232M)	c.1139C > T (p.A380V)	Lin et al. [21]
34	Female	3.56	4.31	c.760C > T (p.R254*)	c.1139C > T (p.A380V)	Lin et al. [21]
35	Female	6.27	3.43	c.695C > T (p.T232M)	c.1139C > T (p.A380V)	Lin et al. [21]
36	Female	2.70	3.46	c.760C > T (p.R254*)	c.51C > G (p.F17L)	Lin et al. [21]
37	Male	7.35	**14.27**	c.338G > A (p.C113Y)	c.338G > A (p.C113Y)	Lin et al. [21]
38	Male	8.25	2.51	c.51C > G (p.F17L)	c.338G > A (p.C113Y)	Lin et al. [26]
39	Male	2.45	1.14	c.760C > T (p.R254*)	c.760C > T (p.R254*)	Lin et al. [26]
40	Male	2.8	1.74	c.760C > T (p.R254*)	c.1161 T > G(p.Y387*)	Lin et al. [26]
41	Female	6.83	4.59	c.760C > T (p.R254*)	c.1400C > G(p.S467C)	Lin et al. [26]
42	Female	6.22	**11.14**	c.760C > T (p.R254*)	c.1400C > G(p.S467C)	Lin et al. [26]
43	Female	6.16	4.02	c.695C > T(p.T232M)	c.1400C > G(p.S467C)	Lin et al. [26]
44	Male	6.77	4.5	c.760C > T (p.R254*)	c.1400C > G(p.S467C)	Lin et al. [26]
45	Female	4.91	6.66	c.250 T > A(p.Y84N)	c.1400C > G(p.S467C)	Lin et al. [26]
46	Male	3.24	4.05	c.51C > G (p.F17L)	c.1196G > A(p.R399Q)	Lin et al. [26]
47	Male	4.96	5	c.51C > G (p.F17L)	c.1195C > T(p.R399W)	Lin et al. [26]
48	Female	3.15	4.09	c.760C > T (p.R254*)	c.1400C > G(p.S467C)	Lin et al. [26]
49	Female	4.12	1.29	c.760C > T (p.R254*)	c.760C > T (p.R254*)	Lin et al. [26]

The C0 levels within the cut-off value are given in bold

C0: free carnitine detected at newborn screening, C0-F1: C0 retested at recall stage, cutoff value: 8.5–50 μmol/L

### *SLC22A5* variants and allele distributions

Forty-nine patients with biallelic pathogenic variants in the *SLC22A5* gene were eventually diagnosed with PCD. A total of 19 distinct *SLC22A5* variants were detected in these 49 patients. Of these, 63.2% (12/19) were missense, 21.1% (4/19) were nonsense, 10.5% (2/19) were frameshift variants, and 5.3% (1/19) affected splicing. All variants have been previously reported, and most of the detected variants were clustered in exons 1, 4, and 7. The most common variant was c.760C > T (p.R254*) with an allele frequency of 31.63%, followed by c.51C > G (p.F17L) (17.35%) and c.1400C > G (p.S467C) (16.33%). The other relatively common variants were c.695C > T (p.T232M), c.338G > A (p.C113Y), c.1139C > T (p.A380V), c.797C > T (p.P266L), and c.1195C > T (p.R399W). Together, these eight variants accounted for 86.73% (85/98) of all mutated alleles (Table 2).

**Table 2:** *SLC22A5* variants and allele distributions in patients with primary carnitine deficiency (PCD)

No	Location	Variants	Variant type	Mutant allele (No.)	Allele frequency (%)
1	Exon 4	c.760C > T (p.R254*)	Nonsense	31	31.63
2	Exon 8	c.1400C > G (p.S467C)	Missense	16	16.33
3	Exon 1	c.51C > G (p.F17L)	Missense	17	17.35
4	Exon 4	c.695C > T (p.T232M)	Missense	7	7.14
5	Exon 1	c.338G > A (p.C113Y)	Missense	4	4.08
6	Exon 7	c.1139C > T (p.A380V)	Missense	4	4.08
7	Exon 4	c.797C > T (p.P266L)	Missense	3	3.06
8	Exon 7	c.1195C > T (p.R399W)	Missense	3	3.06
9	Exon 2	c.428C > T (p.P143L)	Missense	2	2.04
10	Exon 7	c.1161 T > G (p.Y387*)	Nonsense	2	2.04
11	Exon 1	c.250 T > A (p.Y84N)	Missense	1	1.02
12	Intron 1	c.394-1G > A	Splice	1	1.02
13	Exon 4	c.782_799del (p.V261_P266del)	Frameshift	1	1.02
14	Exon 7	c.1144_1162del (p.V382Cfs*45)	Frameshift	1	1.02
15	Exon 4	c.822G > A (p.W274*)	Nonsense	1	1.02
16	Exon 5	c.844C > T (p.R282*)	Nonsense	1	1.02
17	Exon 5	c.845G > A (p.R282Q)	Missense	1	1.02
18	Exon 7	c.1160A > G (p.Y387C)	Missense	1	1.02
19	Exon 7	c.1196G > A (p.R399Q)	Missense	1	1.02
Total				98	100.00

### Relationship between genotype and biochemical phenotype

**Fig. 1:**
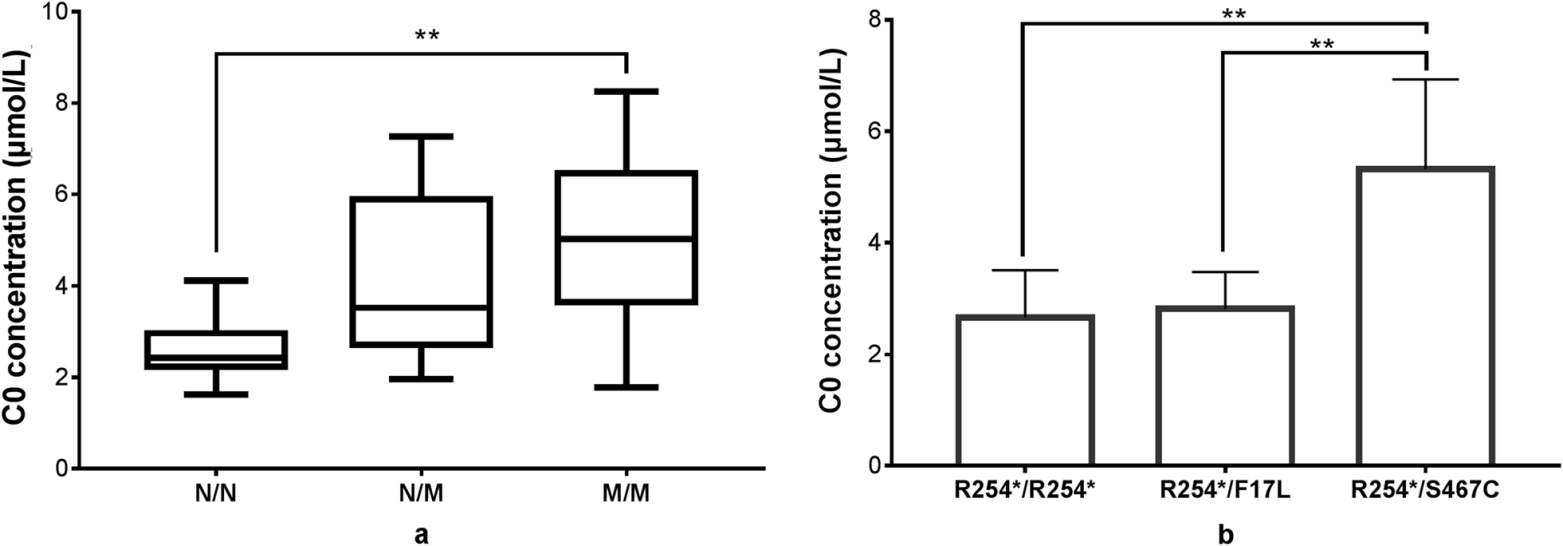
Comparison of the low free carnitine (C0) concentrations (μmol/L) in patients with primary carnitine deficiency (PCD) with different genotypes. N/N: null/null; N/M: null/missense; M/M: missense/missense. Significant differences are indicated by asterisks (*P < 0.05; **P < 0.01; ***P < 0.001) above a bracket connecting two groups

The patients with PCD were divided into three groups: (i) N/N (null/null, n = 8), (ii) N/M (null/missense, n = 22), and (iii) M/M (missense/missense, n = 19). As shown in Fig. 1a, the medians of the N/N, N/M, and M/M groups were 2.43, 3.53, and 5.02, respectively. The C0 level of patients with the N/N genotype was significantly lower than that of the M/M group (*P* = 0.001). Compared to the N/M group, patients with the N/N genotype also had a low C0 level, however, the difference was not significant (*P* = 0.055). The C0 level was not significantly different between the N/M and M/M groups (*P* = 0.304).

Patients with c.760C > T (p.R254*) and two other common variants were grouped. There were 6, 4, and 8 patients with the R254*/R254* genotype, R254*/F17L genotype, and R254*/S467C genotype, respectively. The mean C0 levels in patients with genotypes of R254*/R254*, R254*/F17L, and R254*/S467C were 2.66 ± 0.84, 2.82 ± 0.66, and 5.32 ± 1.61 μmol/L, respectively. The C0 levels of patients with genotypes of R254*/R254* and R254*/F17L were far lower than those of patients with the R254*/S467C genotype (*P* = 0.001 and *P* = 0.005, respectively) (Fig. 1b).

## Discussion

PCD is the most common fatty acid metabolic disorder in China. NBS for PCD has been successfully implemented in most parts of China, and patients usually have an improved prognosis following early L-carnitine therapy. However, NBS for PCD is loaded with a high rate of false-positive results, which appears to be inevitable because the C0 concentration at birth is easily affected by maternal and other factors. Our data showed that NBS for PCD had a high false-positive rate (97.14%) and a low positive predictive value (2.86%), which is very similar to that reported in Taiwan (3%) [14] and California (4.7%) [15]. The incidence of PCD varies greatly throughout China, ranging from 1:8938 to 1:48 717 [13, 16, 17]. The largest-scale NBS study in China showed that the incidence of PCD was 1:30 182 in Zhejiang Province [7]. This study presented more than 500 000 NBS data, indicating that the latest incidence in the Quanzhou area was 1:11 189, which is similar to the incidence recently reported in Guangzhou (1:13 345) and Ningbo (1:16 595) areas [17, 18]. It is noteworthy that the actual incidence of PCD should be higher than reported because the current MS/MS-based NBS has poor sensitivity [19, 20].

NBS results showed that all 49 patients had low C0 levels, although some patients may have normal C0 levels during recall review. These patients were excluded from conventional NBS but could be identified by incorporating second-tier genetic screening [21]. However, it is noteworthy that patients with PCD are also easily missed during NBS because the C0 levels at birth could be affected by the maternal concentration, and therefore, cannot reflect the true levels of the newborns themselves. Luo et al. [22] recently conducted a pilot study in which genetic screening was performed on only 1127 neonates using targeted next-generation sequencing (NGS), and one PCD case of false-negative (C0 = 11.6 μmol/L) was identified. Therefore, NBS for PCD faces challenges, and combining NBS with genetic testing is crucial to improve screening efficiency.

This study updated the *SLC22A5* variant spectrum in a southern Chinese population. The top eight variants together had an allele frequency of 86.73%, which provided important evidence for the rapid screening of targeted *SLC22A5* variants in the Chinese population. Many studies have shown that c.51C > G (p.F17L), c.760C > T (p.R254*), and c.1400C > G (p.S467C) are the three most common variants in the Chinese population, but the variant with the highest frequency varies among diverse regions. For instance, c.1400C > G (p.S467C) was the most frequent variant in the Jining, Suzhou, Guangzhou, Xuzhou, and Ningbo areas [12, 17, 18, 23, 24], and c.51C > G (p.F17L) was the most common variant in the Liuzhou area [25]. In contrast, our data revealed that c.760C > T (p.R254*) was the most common variant in this cohort of patients, and its allele frequency was almost equal to the sum of the other two variants. c.760C > T (p.R254*) is a loss-of-function variant that can cause severe clinical symptoms, which is common in southern China but rarely detected in northern China [14, 16, 24, 26, 27], indicating that this variant presented different geographic distributions. Although c.1400C > G (p.S467C) with residual function may result in a mild phenotype, it was common in both southern and northern China [14, 16, 24, 26], suggesting that this variant is common in the general Chinese population.

Regarding the relationship between genotype and biochemical phenotype, significant differences were observed in C0 levels between patients with N/N and M/M genotypes, and compared to the N/M group, most patients with N/N genotype had low C0 levels, indicating that patients with null variants were associated with low C0 levels. Notably, patients with the N/M genotype may also have very low C0 levels if the missense variant has markedly impaired transport activity. As demonstrated in Fig. 1b, no significant difference was observed in the C0 levels between patients with genotypes of R254*/R254* and R254*/F17L. In contrast, a significant difference was observed in C0 levels between patients with genotypes of R254*/R254* and R254*/S467C because c.1400C > G (p.S467C) retained residual carnitine transport activity.

A major limitation of this study was that patients were diagnosed solely using exome sequencing that focused on the coding regions of the targeted genes. Therefore, disease-causing pathogenic variants in regulatory regions or deep introns, as well as large deletions or duplications, cannot be detected using targeted NGS. In particular, the recently identified c.-149G > A variant in the 5 untranslated region (UTR) of *SLC22A5* was regarded as a frequent cause of PCD, but it was not covered by our targeted NGS. According to the diagnostic criteria of this study, newborns with one or no *SLC22A5* variants were automatically classified as not affected, and a small number of newborns may actually have PCD but not come to our attention. Therefore, more patients may be positive for PCD, and the actual incidence should be higher than reported.

## Conclusions

In summary, this study presented more than 500 000 NBS data with the latest incidence of 1:11 189 in the Quanzhou area. The *SLC22A5* variant spectrum in the selected southern Chinese population was updated, and the top eight variants together had an allele frequency of 86.73%, and c.760C > T (p.R254*) was the most common variant. Patients with null variants were associated with low C0 levels. Combining NBS with genetic testing is critical to improve screening efficiency because patients with PCD may have normal C0 levels during NBS and recall review.

## Methods

### Study population

From January 2014 to June 2021, a total of 548 247 newborns (309 684 men and 238 563 women) who were born in Quanzhou, Fujian Province, China, were screened using MS/MS at the NBS center at Quanzhou Maternity and Children's Hospital. All screen-positive newborns and patients genetically diagnosed with PCD (compound heterozygous or homozygous for *SLC22A5* pathogenic variants) were recruited for this study. This study was approved by the Ethical Committee of Quanzhou Maternity and Children’s Hospital. Written informed consent was obtained from the parents of all patients.

### NBS and diagnostic evaluation

Specimen collection, delivery, and laboratory testing for NBS were performed as previously described [26]. Newborns with low C0 levels (C0 < 8.5 μmol/L, cut-off value: 8–50 μmol/L) were called back. Newborns who tested positive on the second screen were referred for further genetic analysis. Between January 2017 and December 2018, a second-tier newborn genetic screening program targeting 17 *SLC22A5* variants was added to our NBS program [21]. Targeted NGS was performed to capture the exome of 94 known genes relevant to inherited metabolic disorders, as previously described [28]. Individuals with abnormal C0 levels and biallelic pathogenic variants in *SLC22A5* were defined as PCD cases. Individuals with one or no pathogenic variants of *SLC22A5* were excluded from this study.

### Statistical analyses

All statistical analyses were performed using SPSS 26.0 (SPSS Inc., Chicago, IL, USA). Data are expressed as median ± standard deviation when normally distributed, and as median (interquartile range) when non-normally distributed. One-way ANOVA and non-parametric tests were used for the statistical comparisons. Differences between groups were considered significant when *P* < 0.05, *P < 0.05, **P < 0.01, ***P < 0.001).

## Data Availability

The datasets used and/or analysed during the current study can be obtained from the corresponding author upon a reasonable request.

## Abbreviations

PCD: Primary carnitine deficiency
OCTN2: Organic cation transporter type 2
NBS: Newborn screening
MS/MS: Tandem mass spectrometry
C0: Free carnitine
NGS: Next-generation sequencing
N/N: Null/null
N/M: Null/missense
M/M: Missense/missense
