# Correction to: Biochemical and genetic characteristics of patients with primary carnitine deficiency identified through newborn screening

**DOI:** 10.1186/s13023-022-02173-4

**Published:** 2022-01-24

**Authors:** Yiming Lin, Bangbang Lin, Yanru Chen, Zhenzhu Zheng, Qingliu Fu, Weihua Lin, Weifeng Zhang

**Affiliations:** 1 Center of Neonatal Disease Screening, Quanzhou Maternity and Children’s Hospital, 700 Fengze Street, Quanzhou, 362000 Fujian Province China; 2 Administrative Office, Quanzhou Maternity and Children’s Hospital, 700 Fengze Street, Quanzhou, 362000 Fujian Province China; 3 Department of Neonatology, Quanzhou Maternity and Children’s Hospital, 700 Fengze Street, Quanzhou, 362000 Fujian Province China

## Correction to: Orphanet Journal of Rare Diseases (2021) 16:503 https://doi.org/10.1186/s13023-021-02126-3

Following the publication of the original article [1] the authors reported an error in Table 1 (page 3 of the PDF).

**Table 1:** Biochemical and genetic characteristics of 49 patients with primary carnitine deficiency (PCD)

Patient no.	Gender	C0	C0-F1	Genotype		References
1	Male	4.61	6.29	c.51C>G (p.F17L)	c.1195C>T (p.R399W)	This study
2	Female	3.28	3.17	c.51C>G (p.F17L)	c.51C>G (p.F17L)	This study
3	Female	2.37	1.05	c.338G>A (p.C113Y)	c.760C>T (p.R254*)	This study
4	Male	7.65	5.04	c.51C>G (p.F17L)	c.1400C>G (p.S467C)	This study
5	Male	3.75	3.19	c.760C>T (p.R254*)	c.1400C>G (p.S467C)	This study
6	Male	2.72	2.86	c.844C>T (p.R282*)	c.1400C>G (p.S467C)	This study
7	Female	2.54	2.29	c.695C>T (p.T232M)	c.760C>T (p.R254*)	This study
8	Male	5.29	6.58	c.51C>G (p.F17L)	c.1195C>T (p.R399W)	This study
9	Female	5.02	5.53	c.428C>T (p.P143L)	c.428C>T (p.P143L)	This study
10	Female	1.63	1.67	c.760C>T (p.R254*)	c.760C>T (p.R254*)	This study
11	Male	2.31	2.76	c.51C>G (p.F17L)	c.1161T>G (p.Y387*)	This study
12	Female	3.49	3.52	c.51C>G (p.F17L)	c.760C>T (p.R254*)	Lin et al. 2020 [10]
13	Male	1.96	1.73	c.51C>G (p.F17L)	c.760C>T (p.R254*)	Lin et al. 2021 [21]
14	Female	2.40	1.44	c.760C>T (p.R254*)	c.760C>T (p.R254*)	Lin et al. 2021 [21]
15	Male	5.78	**10.67**	c.760C>T (p.R254*)	c.797C>T (p.P266L)	Lin et al. 2021 [21]
16	Male	5.95	**8.64**	c.695C>T (p.T232M)	c.1160A>G (p.Y387C)	Lin et al. 2021 [21]
17	Female	7.27	6.66	c.760C>T (p.R254*)	c.797C>T (p.P266L)	Lin et al. 2021 [21]
18	Female	5.58	5.59	c.760C>T (p.R254*)	c.1400C>G (p.S467C)	Lin et al. 2021 [21]
19	Female	5.34	6.02	c.797C>T (p.P266L)	c.394-1G>A	Lin et al. 2021 [21]
20	Female	1.78	1.90	c.695C>T (p.T232M)	c.1139C>T (p.A380V)	Lin et al. 2021 [21]
21	Male	4.34	4.45	c.51C>G (p.F17L)	c.51C>G (p.F17L)	Lin et al. 2021 [21]
22	Female	4.75	4.16	c.760C>T (p.R254*)	c.845G>A (p.R282Q)	Lin et al. 2021 [21]
23	Female	3.45	5.24	c.760C>T (p.R254*)	c.1400C>G (p.S467C)	Lin et al. 2021 [21]
24	Female	6.82	5.02	c.760C>T (p.R254*)	c.1400C>G (p.S467C)	Lin et al. 2021 [21]
25	Male	2.19	2.12	c.822G>A (p.W274*)	c.782_799del ((p.V261_P266del)	Lin et al. 2021 [21]
26	Male	2.73	**9.84**	c.51C>G (p.F17L)	c.1144_1162del (p.V382Cfs*45)	Lin et al. 2021 [21]
27	Male	3.00	**10.81**	c.51C>G (p.F17L)	c.1400C>G (p.S467C)	Lin et al. 2021 [21]
28	Male	6.46	5.10	c.695C>T (p.T232M)	c.1400C>G (p.S467C)	Lin et al. 2021 [21]
29	Male	3.02	1.77	c.760C>T (p.R254*)	c.760C>T (p.R254*)	Lin et al. 2021 [21]
30	Female	6.77	**10.05**	c.1400C>G (p.S467C)	c.1400C>G (p.S467C)	Lin et al. 2021 [21]
31	Female	2.36	1.75	c.760C>T (p.R254*)	c.760C>T (p.R254*)	Lin et al. 2021 [21]
32	Female	3.12	2.88	c.760C>T (p.R254*)	c.51C>G (p.F17L)	Lin et al. 2021 [21]
33	Male	3.64	3.80	c.695C>T (p.T232M)	c.1139C>T (p.A380V)	Lin et al. 2021 [21]
34	Female	3.56	4.31	c.760C>T (p.R254*)	c.1139C>T (p.A380V)	Lin et al. 2021 [21]
35	Female	6.27	3.43	c.695C>T (p.T232M)	c.1139C>T (p.A380V)	Lin et al. 2021 [21]
36	Female	2.70	3.46	c.760C>T (p.R254*)	c.51C>G (p.F17L)	Lin et al. 2021 [21]
37	Male	7.35	**14.27**	c.338G>A (p.C113Y)	c.338G>A (p.C113Y)	Lin et al. 2021 [21]
38	Male	8.25	2.51	c.51C>G (p.F17L)	c.338G>A (p.C113Y)	Lin et al. 2019 [26]
39	Male	2.45	1.14	c.760C>T (p.R254*)	c.760C>T (p.R254*)	Lin et al. 2019 [26]
40	Male	2.8	1.74	c.760C>T (p.R254*)	c.1161T>G (p.Y387*)	Lin et al. 2019 [26]
41	Female	6.83	4.59	c.760C>T (p.R254*)	c.1400C>G (p.S467C)	Lin et al. 2019 [26]
42	Female	6.22	**11.14**	c.760C>T (p.R254*)	c.1400C>G (p.S467C)	Lin et al. 2019 [26]
43	Female	6.16	4.02	c.695C>T (p.T232M)	c.1400C>G (p.S467C)	Lin et al. 2019 [26]
44	Male	6.77	4.5	c.760C>T (p.R254*)	c.1400C>G (p.S467C)	Lin et al. 2019 [26]
45	Female	4.91	6.66	c.250T>A (p.Y84N)	c.1400C>G (p.S467C)	Lin et al. 2019 [26]
46	Male	3.24	4.05	c.51C>G (p.F17L)	c.1196G>A (p.R399Q)	Lin et al. 2019 [26]
47	Male	4.96	5	c.51C>G (p.F17L)	c.1195C>T (p.R399W)	Lin et al. 2019 [26]
48	Female	3.15	4.09	c.760C>T (p.R254*)	c.1400C>G (p.S467C)	Lin et al. 2019 [26]
49	Female	4.12	1.29	c.760C>T (p.R254*)	c.760C>T (p.R254*)	Lin et al. 2019 [26]

The C0 levels within the cut-of value are given in bold

C0: free carnitine detected at newborn screening, C0-F1: C0 retested at recall stage, cutoff value: 8.5–50 μmol/L

References 19 and 24 in the Table should be renumbered as 21 and 26 respectively.

The correct Table 1 is included in this Correction. The original article has been revised accordingly.
